# Supplementary material for: Fluorination of Terminal Groups Promoting Electron Transfer in Small Molecular Acceptors of Bulk Heterojunction Films
Source: Molecules. 2022 Dec 18;27(24):9037. doi: 10.3390/molecules27249037 (PMC9780906; doi:10.3390/molecules27249037)
Supplement: Supplementary file 1 [file molecules-27-09037-s001.zip › molecules-2006395-supplementary.pdf]

Supporting information

Fluorination of Terminal Groups Promoting Electron Transfer in Small Molecular Acceptors of Bulk Heterojunction Films

Tao Chen[1], Rui Shi[1], Ruohua Gui[1], Haixia Hu[1], Wenqing Zhang[1], Kangning Zhang[1], Bin Cui[1], Hang Yin[1*], Kun Gao[1], Jianqiang Liu[1,2*]

[1] School of Physics, Shandong University, Jinan, 250100, China.
[2] School of Physics and Electrical Engineering, Kashgar University, Kashgar, 844000, China
* Correspondence: hyin@sdu.edu.cn, jqliu@sdu.edu.cn

Fabrication of hole-only device

The hole-only devices were fabricated with a configuration of ITO/PEDOT:PSS/ BHJ/spiro-TBD/Au (Figure S8b). The ITO substrates were cleaned in deionized water, acetone, ethanol, and isopropanol with ultrasonic treatment for 20 min. These ITO substrates were then treated by UV-ozone for 15 min to improve their work function. PEDOT:PSS was spin-coated on ITO substrates with 4000 rpm for 50 s and then annealed at 150 °C for 15 min in air. Subsequently, PM6:Y6 (1:1 wt%, 24mg/ml) was dissolved by heating and stirring at 40 °C in chlorobenzene (12 hours). Then the dissolved solution was spin-coated onto the ITO substrates in a nitrogen-filled glove box, and the spin-coated active layer was annealed at 100 °C for 10 minutes. Finally, spiro-TPD (8nm) and Au (80nm) were deposited by thermal evaporation.

The Caculation of Crystalline Coherence Length

GIWAXS characterizes the molecular packing and crystallinity of the active layer. The d-spacing associated with the π-π stacking peak indicates the molecular interlayer spacing, which can be calculated from the following equation:

$$d = \frac{2\pi}{q_{z(010)}}$$

In which, $q_{z(010)}$ is the position of the π-π stacking peak in Q space. We further calculated the crystalline coherence length (CCL) using the Scherrer equation and quantitatively compared the crystallinity of the blend films:

$$CCL = \frac{2\pi k}{FWHM}$$

In which, k is the shape factor (0.9) and $FWHM$ is the full width at half maximum of the π-π stacking peak.

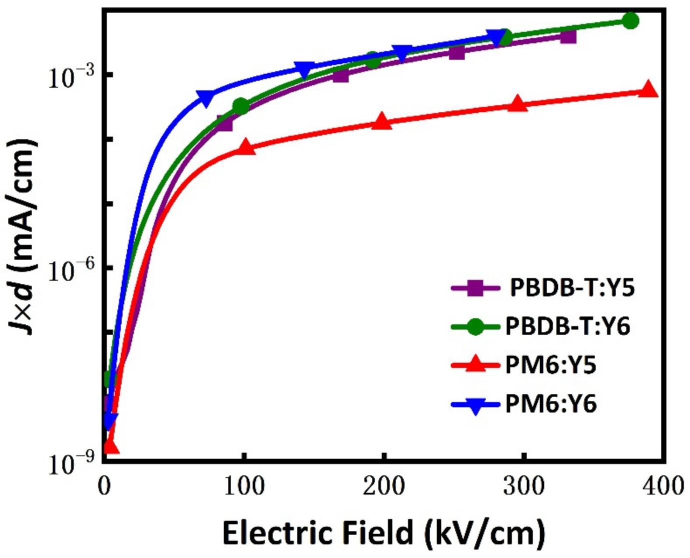

Figure S1. Hole carrier transport results for the PBDB-T:Y5, PBDB-T:Y6, PM6:Y5 and PM6:Y6 BHJ films: $J \times d$ as a function of the applied electric field.

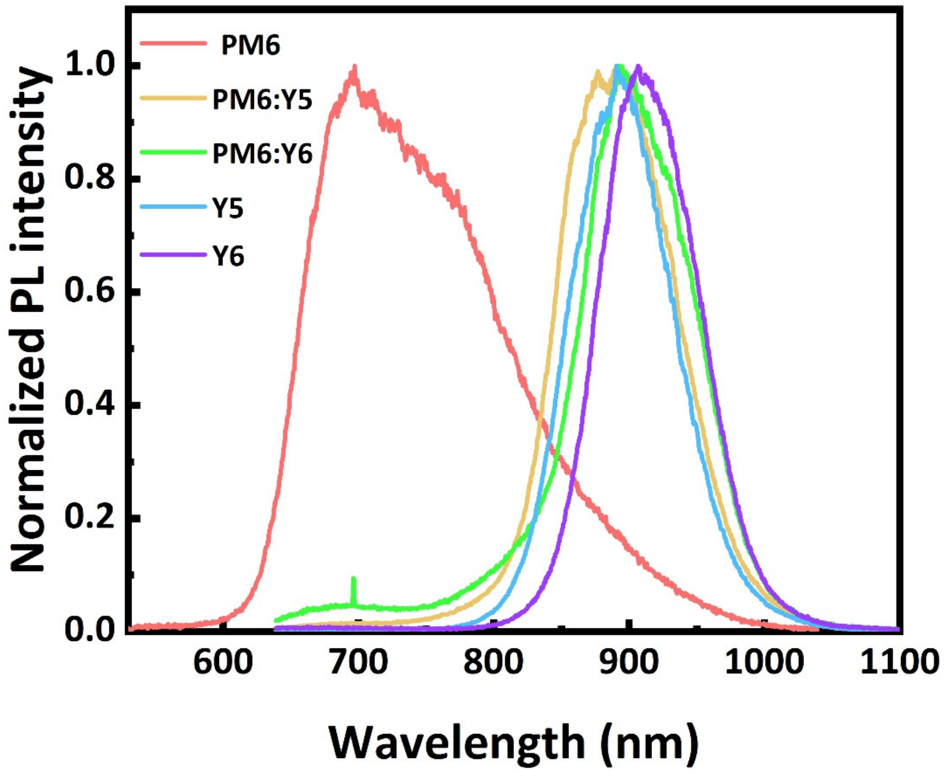

Figure S2. Normalized PL spectra of the PM6, Y5, Y6 and their blend films. The films were excited

at 400nm.

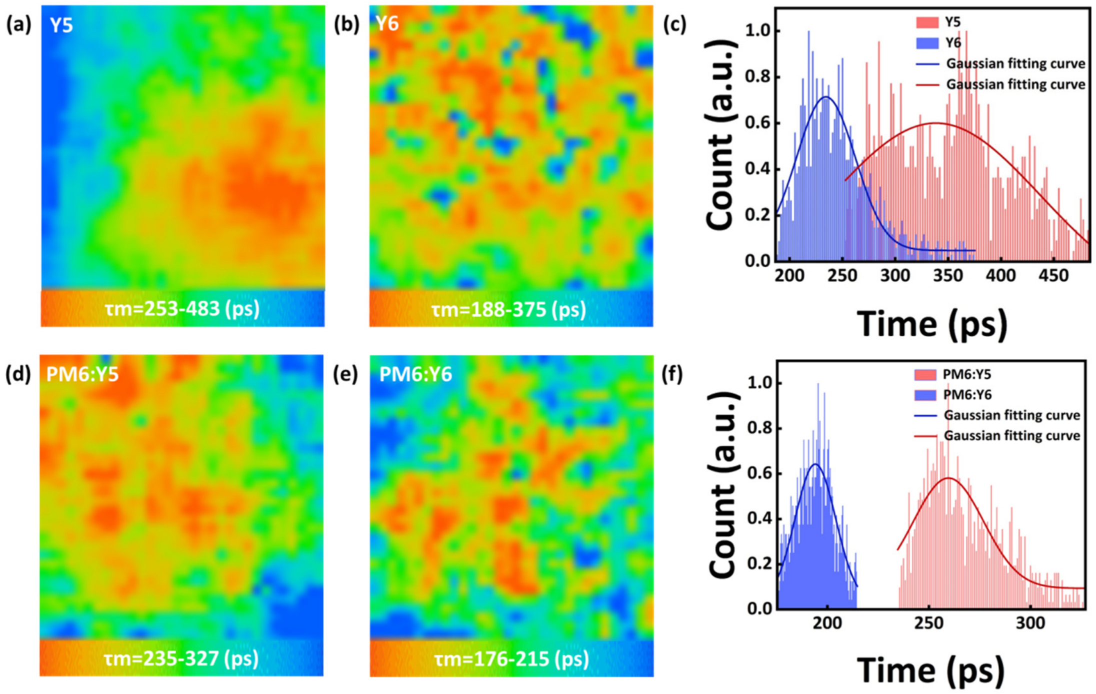

Figure S3. 2D time-resolved fluorescence images (10 μm ×10 μm) of (a) neat Y5, (b) neat Y6 and (d) PM6:Y5, (e) PM6:Y6 blend films and [redacted] the corresponding distribution histogram of exciton lifetime as a function of storage time.

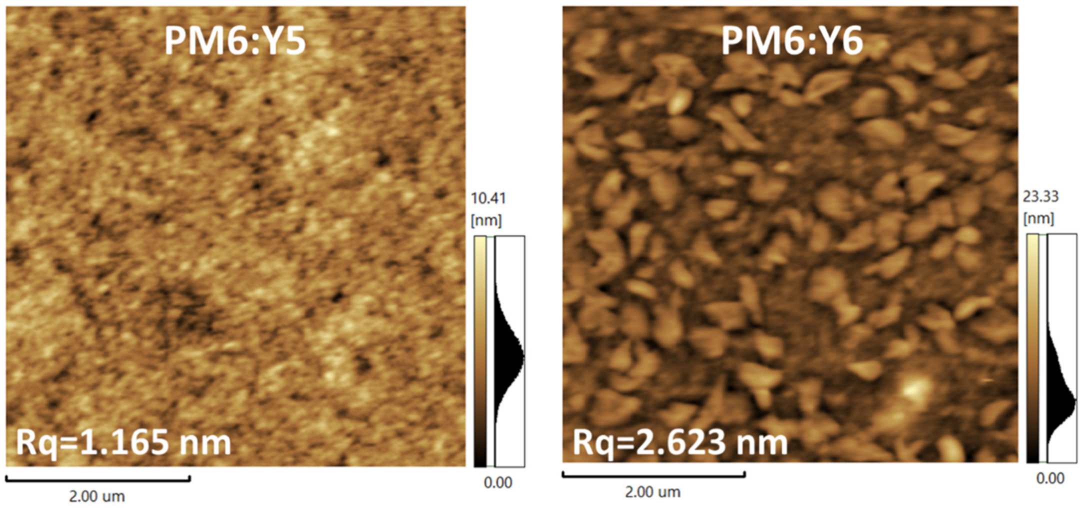

Figure S4. Morphology images (5μm × 5μm) of the blend films: AFM height images of PM6:Y5 and PM6:Y6 BHJ films.

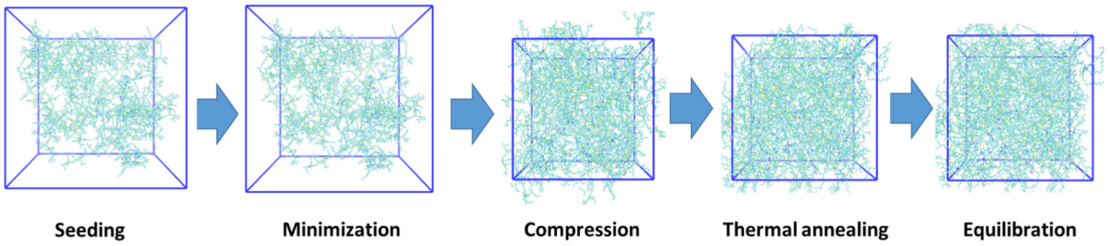

Figure S5. The procedure of molecular dynamics (MD) simulations from molecular seeding to equilibration of the molecular systems, taken PM6:Y6 as an example.

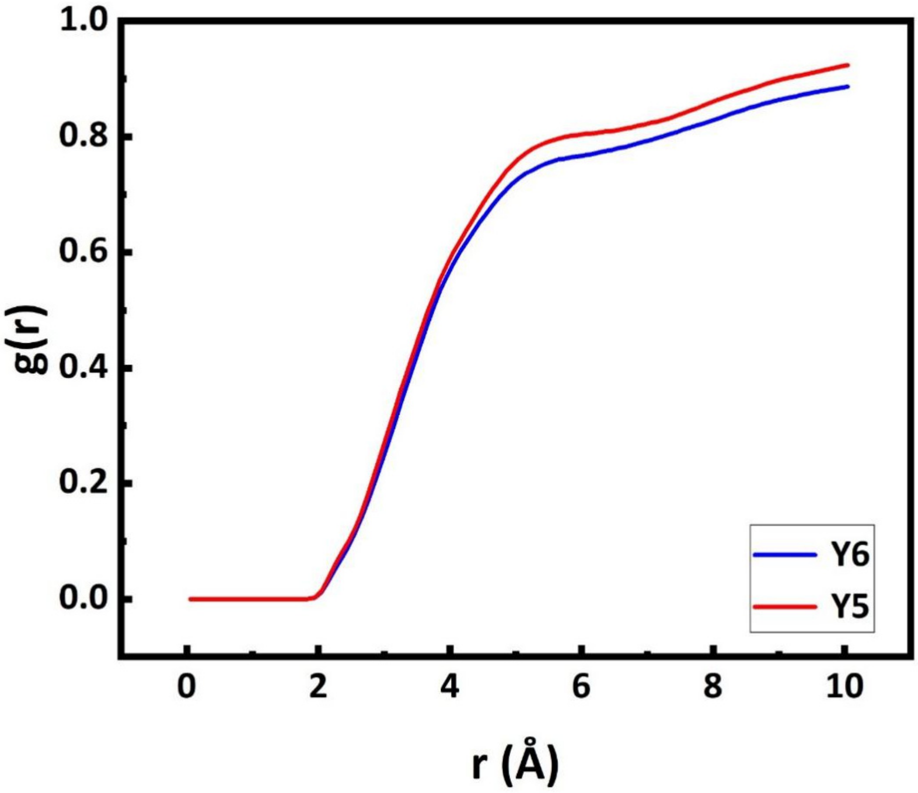

Figure S6. Radial distribution functions (RDFs) for Y6 in PM6:Y6 film and Y5 in PM6:Y5 film.

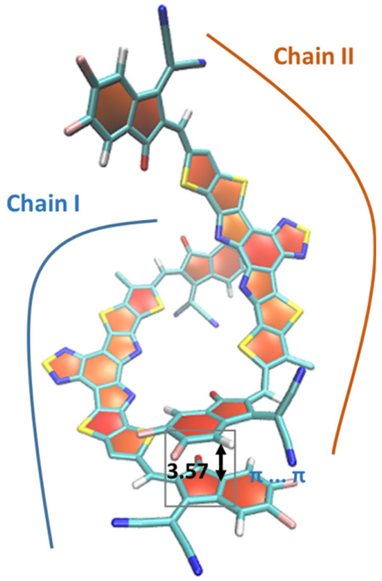

Figure S7. Representative configurations of Y6 dimer of "A-to-A" type J-aggregates.

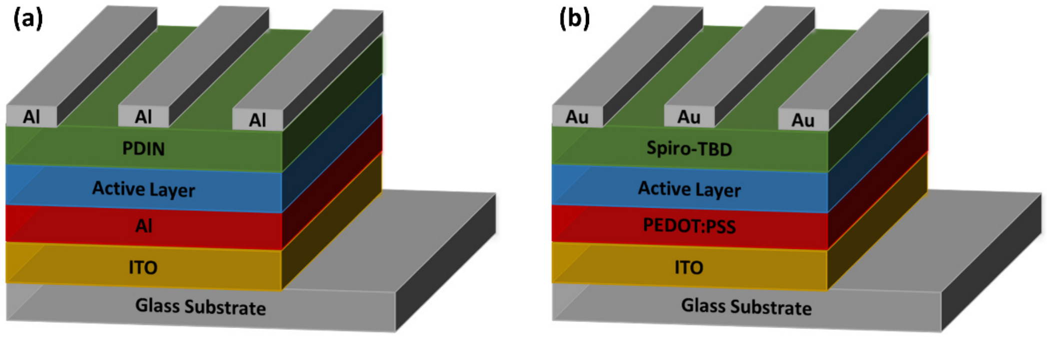

Figure S8. Schematic diagram of (a) the electron-only device and (b) the hole-only device mentioned in this work.

Table S1. A summary of charge transport parameters of PM6:Y6, PM6:Y5, PBDB-T:Y6, and PBDB-T:Y5 BHJ films.

Device	$\mu_e/10^{-5}$ cm^2 v^{-1} s^{-1}	$\mu_h/10^{-5}$ cm^2 v^{-1} s^{-1}	σ_e/meV
PM6:Y6	5.02	35.4	47
PM6:Y5	0.0576	2.79	59
PBDB-T:Y6	1.04	12.6	57
PBDB-T:Y5	2.05	9.69	63
